# Synergistic effects of multiword sequences structure, function, frequency and association on raters’ evaluations of essay quality

**DOI:** 10.3389/fpsyg.2022.1026658

**Published:** 2022-10-31

**Authors:** Yuan Ke Li, An Bang Fang

**Affiliations:** South China Normal University, Guangzhou, China

**Keywords:** multiword sequences, structures and functions, statistical features, L2 writing quality, EFL (English as a foreign language) learning

## Abstract

Despite accumulated research findings confirming the link of multiword sequences (MWSs) structures and functions to essay quality, as well as the connection between MWSs statistical features (e.g., their frequency and association strengths in BNC/COCA) and writing quality, to date no study integrated these two separate lines of investigations. It remains to investigate whether and how MWSs structures, functions and their statistical features jointly affect writing quality. Drawing on 900 rated argumentative essays composed by Chinese grade 12 students in National Matriculation Test, the present study employed CollGram to automatically identify the nativelike 4-word sequences in these essays and to analyze their frequency and Mutual Information (MI) scores in COCA. The structures and functions of frequent nativelike 4-word sequences were also analyzed manually. A serial of linear mixed-effect models was constructed to investigate their main effects as well as interaction effects on essay scores. The best fit model revealed the links of higher essay scores to higher MI scores, to more noun-phrase sequences, to more stance sequences, as well as to fewer referential sequences. Additionally, the interaction of prepositional phrase sequences and their frequency in COCA affected essay scores, so did the interaction of verb phrase sequences and their MI in COCA, as well as the interaction of noun phrase sequences and their MI in COCA. The findings provide new insights into the complex interaction between MWSs structures, functions and their statistical features, as well as their joint effects on writing quality.

## Introduction

Multiword sequences (MWSs; e.g., lexical bundles, collocations, binomials) play an important role in first and second language acquisition, processing, fluency, idiomaticity, and instruction (Ellis et al., 2008; O’Donnell et al., 2013; Siyanova-Chanturia and Van Lancker, 2019). In the past two decades, growing corpus-based studies adopted frequency-based approach (Biber et al., 1999) to identify lexical bundle (i.e., frequent contiguous MWSs) in English learner essays, and their research findings have confirmed the link of bundle structures (e.g., clause, noun phrase, verb phrase) and functions (e.g., referential, discourse organizing, stance) to essay quality (Staples et al., 2013; Chen and Baker, 2014; Qin, 2014; Appel and Wood, 2016; Appel, 2022; Kim and Kessler, 2022). Meanwhile, in another more recent line of investigations, researchers employed natural language processing tools (e.g., TAALES, CollGram) to automatically identify the nativelike n-grams (contiguous MWSs) in English essays appearing in British National Corpus (BNC) or Corpus of Contemporary American English (COCA). Statistical analyses in these studies revealed that their statistical features (e.g., frequency and association in BNC/COCA) were associated to raters’ judgement of essay quality (Granger and Bestgen, 2014; Kyle and Crossley, 2016; Yoon, 2016; Kim et al., 2018; Garner et al., 2019; Zhang and Li, 2021).

Notably, however, to date no study integrated these two separate lines of investigations. It remains to investigate whether and how multiword sequences (MWSs) structures, functions and their statistical features jointly affect writing quality. Investigating their synergistic effects on essay quality will shed new light on the complex and dynamic relationships among different features associated to L2 phraseological use and their joint effects on L2 writing quality, as well as advance corpus-based research on phraseology. Drawing on 900 rated argumentative essays composed by Chinese high school EFL learners, in this study we used CollGram (Wołk et al., 2017) to automatically identify all nativelike contiguous 4-word sequences (e.g., *a positive effect on*) in these essays and to analyze their frequency and Mutual Information (MI) scores in COCA. The structures and functions of frequent nativelike 4-word sequences were also analyzed manually. We then employed mixed-effect modeling to investigate their synergistic effects (i.e., the main effects of these features and their interaction effects) on essay scores. The main effects of these features on essay scores are a part of their synergistic effects because their individual effects on essays scores may be affected by other features when they are simultaneously included into one model predicting essay scores. For example, the main effects of sequence structures and functions on essays scores before and after their frequency and MI in COCA were brought into the prediction model may be different, and vice versa.

## Literature review

### The effects of MWSs structures and functions on L2 writing quality

Accumulated learner corpus research investigated lexical bundles (Biber et al., 1999), namely frequent contiguous MWSs (e.g., *I want you to*), in English as a foreign language (EFL) essays at different proficiency levels, and their findings have shown varied quantitative distributions of bundle structures and functions among different levels of essays (Vidakovic and Barker, 2010; Biber and Gray, 2013; Chen and Baker, 2014; Appel and Wood, 2016; Kim and Kessler, 2022). For example, Chen and Baker (2014) analyzed bundles in essays composed by Chinese university EFL learners rated at B1, B2 and C1 levels of CEFR. They found that lower-rated essays contained a higher proportion of verb phrase bundles, whereas higher-rated essays included a greater proportion of noun phrase bundles and prepositional phrase bundles. Appel and Wood (2016) analyzed the functions of bundles in essays composed by Canadian high school students, finding that low-level essays used more stance bundles and more discourse-organizing bundles, whereas high-level essays used more referential bundles. However, mixed research findings existed. Staples et al. (2013) found that the quantitative distributions of stance bundles as well as discourse organizing bundles were similar among TOEFL essays from different proficiency levels.

Additionally, findings from bundle-based research also show that there were substantial differences in specific patterns of structures and functions associated to bundles between higher-rated and lower-rated essays (Chen and Baker, 2014; Qin, 2014; Vo, 2019; Kim and Kessler, 2022). For example, Qin (2014) analyzed non-native graduate student English essays and published research articles in applied linguistics, finding that graduates at higher levels of study used more bundles consisting of *noun phrases with post-modifier* fragments (e.g., *no significant differences among the*) than lower level graduates. These bundles also appeared more in published research articles when compared to graduates’ essays. Additionally, Kim and Kessler (2022) found that several discourse-organizing bundles, such as *first of all*, *what is more*, and *in a word,* appeared more in high-scoring essays composed by Chinese college learners of English, while low-scoring essays contained more bundles expressing stance bluntly (e.g., *you cannot*) and colloquially (e.g., *I think cellphone use should*).

Furthermore, a few research analyzed more flexible MWSs including one or more variable slot such as phrase-frames (*p*-frames) in English learner essays across different proficiency levels. For instance, Garner (2016) investigated 100 most frequent 4-word *p*-frames (e.g., I ^*^ like to) in L1 German learners of English essays from five proficiency levels, respectively, in the EF-Cambridge Open Language Database (EFCAMDAT). His research findings revealed that in higher-rated essays the most frequent 4-word *p*-frames were more variable, less predictable, and more functionally diverse. Tan and Römer (2022) analyzed 3-word *p*-frames (e.g., the ^*^ is) and 4-word *p*-frames in rated essays composed by L1 Chinese English learners across different proficiency levels in EFCAMDAT corpus. Like Garner (2016), their research findings also supported that in higher-rated essays the frequent *p*-frames were more variable and less predictable.

### The effects of MWSs statistical features on L2 writing quality

Informed by usage-based research finding that input frequency and association were linked to construction acquisition and production (Ellis et al., 2008, 2016), an emerging line of L2 writing research investigated the effect of statistical features (e.g., frequency, association, range, etc) of n-grams (contiguous MWSs) employed by L2 writers on raters’ evaluation of their essay quality. In an early study of this strand, Granger and Bestgen (2014) analyzed bigrams in intermediate (CEFR B1 and B2) and advanced (CEFR C1 and C2) English essays, finding that intermediate essays contained more high frequency bigrams in BNC but fewer low-frequency and strongly associated bigrams (attested by their MI) in BNC when compared to advanced essays. Bestgen and Granger (2014) used CollGram to extract bigrams from English essays in Michigan State University Corpus and to assign to each extracted bigram mutual information (MI) and *t*-score computed on the basis of COCA, and investigated their relationship to L2 writing development and text quality assessment. They found that in students’ essays bigrams made up of high-frequency words in COCA decreased over time, and that higher essay scores were linked to higher mean MI of bigrams in COCA, and to lower proportion of bigrams that were absent from COCA, most of which were erroneous.

Recently, drawing on a corpus of English placement test essays written by L1 Korean learners of English, Garner et al. (2019) employed TAALES 2.0 (Kyle and Crossley, 2015) to automatically identify the nativelike bigrams and trigrams in these essays that also appear in COCA, and to generate multiple indices associated to their frequency, range, association strength in the Academic and Spoken sub-corpora of COCA. Results of multiple regression analyses revealed that higher rated essays included more strongly associated academic bigrams, a greater proportion of frequent academic trigrams, and more strongly associated spoken trigrams. Zhang and Li (2021) replicated Garner et al. (2019) study. Zhang and Li (2021) found that the quality ratings of 1,643 expository essays composed by Chinese college English learners were predicted by spoken bigram proportion, the directional association strength of spoken trigrams, mutual association strength of spoken bigrams, and spoken bigram range.

Additionally, the research findings of several studies investigating specific types of combinations (e.g., verb-nouns combinations, adjective-nouns combinations) also confirmed the link of their frequency and association to essay scores (Yoon, 2016; Paquot, 2019; Garner, 2022). Paquot (2019) investigated to what extent measures of phraseological complexity (sophistication and diversity) associated to adjective-noun (amod), adverb-adjective (advmod) and verb-direct object (dobj) combinations discriminated rated proficiency levels of English essays composed by upper-intermediate and advanced (B2, C1 and C2 in the CEFR) French EFL college students who majored in linguistics. Phraseological sophistication was operationalized in two ways, namely (1) ratio of these three types of combinations pertaining to academic collocations included in Academic Collocations List (Ackermann and Chen, 2013), and (2) the average pointwise mutual information (MI) score for these three types of combinations in a large reference corpus of published L2 research articles. Phraseological diversity, the second dimension of phraseological complexity, was operationalized as root type-token ratio (RTTR) computed for each type of combinations. The results show that phraseological diversity associated to these three types of combinations and ratio of these three types of combinations pertaining to academic collocations did not significantly discriminate proficiency levels, but mean MI scores for all three types of combinations did. Moreover, the MI-based association measures for the three types of combinations performed better than syntactic complexity measures and lexical complexity measures to gauge essay ratings. Garner (2022) investigated verb-noun combinations in TOEFL essays across different proficiency levels, finding that higher-rated essays not only contained a more diverse range of verb–noun combinations, but also included more verb–noun combinations that were less frequent but more strongly associated in COCA.

Furthermore, a few studies analyzed verb argument constructions (VAC), which consist of a verb slot and the related arguments in a syntactic construction, in learner essays (Kyle and Crossley, 2017; Römer, 2019; Mostafa and Crossley, 2020). Kyle (2016) developed the Tool for Automatic Analysis of Syntactic Sophistication and Complexity (TAASSC) to analyze fine-grained clausal, noun phrase and VAC indices. The current version (1.3.8) generated 190 VAC-based indices on the basis of COCA (e.g., frequency of main verb lemmas, strength of association between main verb lemma and VACs). Kyle and Crossley (2017) investigated the relationship of these VAC indices to TOEFL independent essay scores, finding that higher-scoring essays contained less frequent and more strongly associated verb-VAC combinations. Mostafa and Crossley (2020) investigated the relationship of VAC-based indices to rated scores of descriptive essays, independent essays as well as integrated essays, finding that the significant VAC-based indices predictive of essay scores varied among the three types of essays.

In short, despite accumulated research findings have confirmed the link of essay scores to MWSs structures and functions, as well as to their statistical features (e.g., frequency and MI), less is known about their synergistic effects, namely (1) to what extent these features affect essay scores when they are simultaneously included in a model predicting essay ratings, and (2) whether there are any interactions between MWSs structures or functions and their statistical features, and if so, how their interactions affect raters’ evaluation of essay quality. It is important to investigate these questions due to the following reasons. The research findings from the first question will provide useful insights into which features associated to multiword sequences in learner essays better predict raters’ evaluation of essay quality when multiple features (structure, function, as well as statistical features) are brought into investigations. The research findings from the second question will provide deeper insights into the complex relationships between the structural and functional characterizations of multiword sequences in essays and their statistical features (their frequency and MI in COCA), as well as reveal their interaction effects (if any) on essay ratings. Moreover, in recent years there have been more investigations into the phraseological use in essays composed by adolescent EFL learners (e.g., Leńko-Szymańska, 2014), but their relationship to raters’ evaluations of writing quality was still understudied. To close these gaps, drawing on a corpus of rated English argumentative essays composed by Chinese high school students in National Matriculation Test, in this study we aimed to advance the thin line of integrated investigations by analyzing the structures and functions of frequent nativelike 4-word sequences (e.g., *a positive effect on*) in these essays as well as their frequency and association (MI) in COCA. We then constructed mixed-effect models using these features to predict essay scores, with a view to investigating the following research questions:

1. What were the relationship of essay scores to frequent nativelike 4-word sequences structures and functions in essays, as well as their frequency in COCA and their MI in COCA?

2. What were the relationship of essay scores to the interactions, if any, of their structures or functions and statistical features (frequency in COCA, MI in COCA)?

## Research methodology

### The corpus of rated argumentative essays

900 rated argumentative essays composed by Chinese grade 12 students in National Matriculation English Test (NMET) in 2014 were analyzed. All essays were written in response to the task in Appendix 1. In all essays there were two parts, as task instructions required. In the first part, students summarized the main ideas of a short English passage about Miss McCarty’s donation of her life savings to help students. This part should contain approximately 30 words. In the second part, students expressed their views on Miss McCarty’s behavior, described the impact of her donation on students, as well as illustrated the beneficiary to whom they would donate money to, if they were to donate, and explained reasons. The second part should contain approximately 120 words.

The corpus consisted of 5,349 word types and 184,761 tokens. It was well-balanced to contain 60 essays from 15 score levels between 10 points and 24 points. Essays rated below 10 were not made available by the test administrator from whom these essays along with their final scores were obtained. Essays scoring the full mark (25 points) were also excluded because they were extremely rare. A large pool of well-trained raters who were experienced high school English teachers with at least 8 years’ teaching experiences were recruited by the test administrator to rate these essay scripts, using a rubric covering (1) using one’s own words to summarize the story, (2) covering all required content elements, (3) lexical resources, (4) grammatical range and accuracy, (5) cohesion and coherence. All raters participated in a required one-hour rater training session offered by the NMET administrator and they passed a qualification test before starting the rating process.

Each of these essays in the corpus was rated blindly and independently by two raters randomly selected from the pool. If the two scores assigned by two independent raters to an essay had a division of three points or lower, their average score was the essay’s final score. On the other hand, if the two scores assigned by two independent raters to an essay had a division of four points or more, the third rater would reassess the essay, in which case the average of the two closest scores was its final score.

### 4-Word sequences extraction and analysis

**Figure 1:**
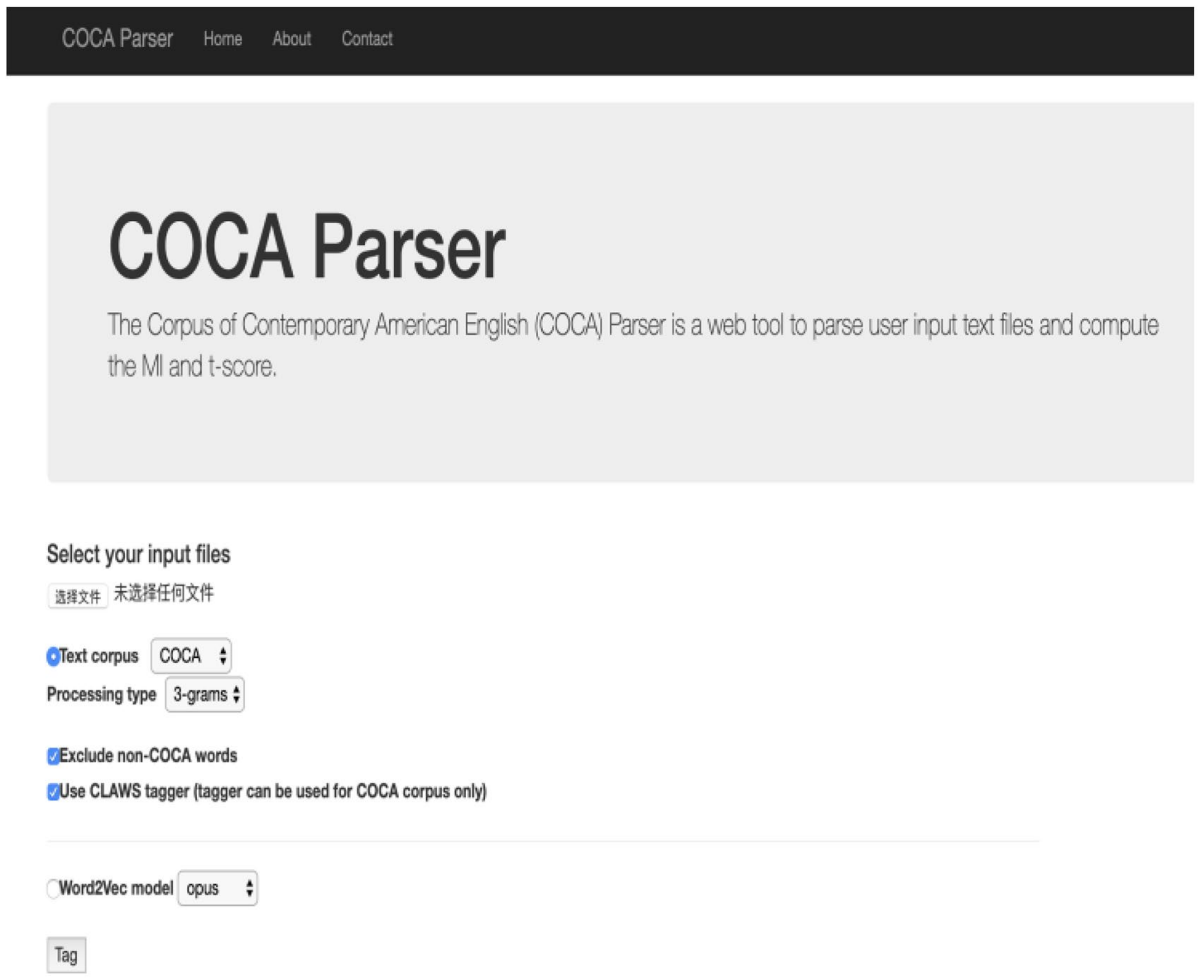
The online interface of CollGram (Reproduced with permission from Wołk et al., 2017).

All essays in the corpus were uploaded to the online natural language processing tool CollGram (Wołk et al., 2017).^1^ The online interface of this tool is shown in Figure 1. It can extract contiguous multiword sequences (i.e., n-gram) consisting of 2, 3 or 4 words appearing once or more in the uploaded essays, analyze which extracted sequences also appear in COCA (Davies, 2009), and assign to them several statistical features such as their frequency in the essays, their frequency in COCA as well as their association (MI, t) in COCA. The version of COCA used in CollGram contained more than 425 million words (20 million words each year from 1990 to 2011) and is equally divided among speech, fiction, popular magazines, newspapers and academic texts (see Bestgen and Granger, 2014, p: 33).

In the current study, we investigated 4-word sequences because among 2-word, 3-word and 4-word sequences that CollGram can automatically extract, 4-word sequences exhibited a clearer range of structures and functions (Hyland, 2008, p: 8). CollGram extracted 1,892 contiguous 4-word sequences (e.g., *a positive effect on*) appearing once or more in the essay corpus. To identify frequent sequences in the essays, we extracted the sequences which appeared at least five times in the corpus as well as in five or more different essays. These two criteria filtered out low frequency sequences (below five occurrences) in the corpus as well as sequences that appeared in a small number of essays (fewer than five essays). 495 sequences met these criteria, and among them 490 sequences appeared in COCA (as indicated by CollGram). These frequent nativelike sequences were the focal items under examination. Their structures and functions were analyzed by the two co-authors independently. For structural coding, we employed Lu and Deng (2019) taxonomy (i.e., noun phrase (NP)-based, prepositional phrase (PP)-based, verb phrase (VP)-based, and clause-based), as it merged Biber et al. (2004) categories and Pan et al. (2016) categories and was therefore more comprehensive. For functional coding, we adopted Biber et al. (2004) taxonomy to classify sequences into three functional types, namely referential (e.g., *a great number of*), stance (e.g., *want to make a*), and discourse organizing (e.g., *on the other hand*). The two coders first conducted an independent coding of the same 5% random sample of sequences, and checked inter-coder reliability through Cohen’s Kappa. Cohen’s Kappa was 0.95 for structural coding, and 0.91 for functional coding, indicating satisfactory inter-coder reliabilities. We coded the remaining sequences separately, and resolved any disagreements through discussion.

### Statistical analysis

Before conducting statistical analyses, standardization of variables were performed (see Murakami and Ellis, 2022) because the independent variables, namely the quantities of different structures and functions associated to frequent nativelike 4-word sequences, as well as their mean frequency in COCA and their mean MI in COCA, had different units of measurements. To this end, all independent variables as well as dependent variable (essay scores) were transformed into their *z* scores and all subsequent analysis were conducted using their *z* scores (see Murakami and Ellis, 2022). Variance inflation factors (VIF) for all independent variables were examined through the “MuMIn” package in R, and the results confirmed that they all had a VIF lower than 5, suggesting absence of multi-collinearity (Shrestha, 2020). After that, Generalized Linear Mixed-effects Modeling (GLMM) with lme4 (Bates et al., 2015)) and lmer Test (Kuznetsova et al., 2017) packages in R (R Core Team, 2015) was employed to investigate the main effects of the extracted nativelike 4-word sequences structure, function, their mean frequency in COCA, their mean MI in COCA as well as their interaction effects on essay scores. Several authors recommended to adopt the maximal model (Murakami, 2016; Duan and Shi, 2021), namely the model containing all possible predictors with lowest Akaike Information Criterion (AIC) scores, which measure the goodness of fit. Therefore, we started by building the unconditional model (Bates, 2010) which included by-subject random intercepts only without including any fixed-effects predictor in the model. To this random effect-only model (model 1), fixed main effects associated to the quantities of different structures and functions of the frequent nativelike 4-word sequences, as well as their mean frequency in COCA and their mean MI in COCA were added one at a time to derive nine more complex models (models 2–10). Additionally, the fixed interactive effects associated to the interaction of their statistical features (frequency in COCA, MI in COCA) and the quantities of their different structures or functions were added to model 10, again one at a time, to derive another 14 models. Altogether, 24 models were constructed, among which the best fit model was identified through AIC-based comparisons as well as maximum likelihood (ML) test (Murakami, 2016). We employed maximum likelihood estimation instead of restricted maximum likelihood (REML) procedures because REML does not allow for the comparison of models with different fixed-effects structure (Bolker et al., 2009), and also because REML estimates are not well defined for GLMMs (Bates, 2009). The best model should have the lowest AIC, and additionally it should also significantly (*p* < 0.05) improve on all other less complex models (see Cunnings and Finlayson, 2015; Gries, 2021). The “hand picking” program in “sjPlot” package of R was employed to draw the simple slope diagram to visualize the significant interactive effects.

## Results

### Model comparisons

Table 1 shows the by-subject random effect-only model (model 1) and 12 more complex models which were generated by adding predictors associated to fixed main effects and three interactions among them. In Table 1, the AIC of these models decreased consistently until model 13, indicating an improved fit of model 13 over the other less complex models. Additionally, ML test also confirmed that model 13 significantly improved on all other less complex models (*p* < 0.05).

**Table 1:** Comparing the best fit model (model 13) to less complex models.

Models	Fixed-effect	Random effects	AIC	ΔAIC (compared to prior model)	ML test against prior model	
					χ^2^	*p*
Model 1	None	By subject	2476.8			
Model 2	structure_PP	By subject	2450.8	−26.0	27.98	<0.001^***^
Model 3	Model2 + structure_NP	By subject	2426.7	−24.1	26.14	<0.001^***^
Model 4	Model3 + structure_VP	By subject	2408.2	−18.5	20.49	<0.001^***^
Model 5	Model4 + structure_CL	By subject	2403.1	−5.1	7.10	<0.01^**^
Model 6	Model5 + function_DO	By subject	2400.3	−2.8	4.74	<0.05^*^
Model 7	Model6 + function_RE	By subject	2394.3	−6.0	8.03	<0.01^**^
Model 8	Model7 + function_SE	By subject	2373.9	−20.4	22.42	<0.001^***^
Model 9	Model8 + frequency	By subject	2371.5	−2.4	4.36	<0.05^*^
Model 10	Model9 + MI	By subject	2251.8	−119.7	121.76	<0.001^***^
Model 11	Model 10+ MI × structure_VP	By subject	2243.5	−8.3	10.25	<0.01^**^
Model 12	Model 11+ MI × structure_NP	By subject	2239.6	−3.9	5.92	<0.05^*^
Model 13	Model 12+ frequency×structure_PP	By subject	2235.6	−4.0	6.00	<0.05^*^

PP (prepositional phrase), NP (noun phrase), VP (verb phrase), CL (clause), DO (discourse organizing), RE (referential expression), SE (stance expression), frequency (mean frequency in COCA), MI (mean Mutual Information in COCA).

* *p* < 0.05;

** *p* < 0.01;

*** *p* < 0.001.

We further added to model 13 additional fixed effects associated to other interactions of statistical features (frequency in COCA, MI in COCA) and structure or functions, resulting in 11 more complex models presented in Table 2. As can be seen, compared to model 13, the more complex models except model 15 had higher AIC scores, indicating that their goodness of fits were not as good as that of model 13. Although model 15 had a lower AIC score than model 13 (ΔAIC = −0.4), the ML test showed that this difference was not significant (*p* > 0.05). As such, model 13 outperformed model 15 because the former was more parsimonious.

**Table 2:** Comparing model 13 to more complex models.

	Fixed-effect	AIC	ΔAIC (compared to model 13)	ML test against model 13	
				χ^2^	*p*
Reference Model	Model 13	2235.6			
*Model 13 + MI × structure*					
Model 14	Model 13 + MI × structure_PP	2237.2	1.6	0.547	>0.05
Model 15	Model 13 + MI × structure_CL	2235.2	−0.4	0.122	>0.05
*Model 13 + frequency × structure*					
Model 16	Model 13 + frequency × structure_NP	2236.2	0.6	0.235	>0.05
Model 17	Model 13 + frequency × structure_VP	2237.6	2.0	0.823	>0.05
Model 18	Model 13 + frequency × structure_CL	2237.5	1.9	0.764	>0.05
*Model 13 + MI × function*					
Model 19	Model 13 + MI × function_DO	2237.5	1.9	0.740	>0.05
Model 20	Model 13 + MI × function_RE	2236.5	0.9	0.303	>0.05
Model 21	Model 13 + MI × function_SE	2237.6	2.0	0.971	>0.05
*Model 13 + frequency × function*					
Model 22	Model 13 + frequency × function_DO	2237.6	2.0	0.883	>0.05
Model 23	Model 13 + frequency × function_RE	2236.3	0.7	0.254	>0.05
Model 24	Model 13 + frequency × function_SE	2237.6	2.0	0.858	>0.05

PP (prepositional phrase), NP (noun phrase), VP (verb phrase), CL (clause), DO (discourse organizing), RE (referential expression), SE (stance expression), frequency (mean frequency in COCA), MI (mean Mutual Information in COCA).

Taken together, model 13 had the best fit among all models. Its effect size was calculated by using the MuMIn package in R through two forms of R square values: marginal (R^2^m = 0.260) and conditional (R^2^c = 0.275). Marginal R^2^ values are associated with only the fixed effects while conditional R^2^ values reflect both the fixed effects and the random effects combined (Wolter and Yamashita, 2018; Siyanova-Chanturia and Spina, 2020). The marginal R^2^ of model 13 indicated that its fixed effects explained 26% variance of essay scores. In Section 4.2 below we will report the predictors in the best fit model, model 13, as well as the significant main effects and interaction effects on essay scores.

### The best model (model 13)

Table 3 presents the fixed effects associated to frequent nativelike 4-word sequences in the best fit model (model 13) which predicted essay scores. It revealed that higher essay scores were linked to their higher mean MI scores (Estimate = 0.427, *t* = 10.958, *p* < 0.001), to more noun phrase sequences (Estimate = 0.077, *t* = 2.139, *p* < 0.05), to more stance sequences (Estimate = 0.100, *t* = 2.751, *p* < 0.01), and to fewer referential sequences (Estimate = −0.107, *t* = −3.054, *p* < 0.01). Additionally, there were several significant interactive effects on essay scores as well. Essay scores were also affected by the interaction of quantities of verb phrase sequences and their MI in COCA (Estimate = −0.068, *t* = −2.395, *p* < 0.05), the interaction of quantities of noun phrase sequences and their MI in COCA (Estimate = −0.065, *t* = −2.161, *p* < 0.05), as well as the interaction of quantities of prepositional phrase sequences and their frequency in COCA (Estimate = −0.072, *t* = −2.445, *p* < 0.05).

**Table 3:** Fixed effects in the best model (model 13).

	Fixed effects:	Estimate(B)	SE	t	Pr(>|t|)
1	(Intercept)	0.068	0.033	2.030	<0.05^*^
2	structure_PP	0.061	0.035	1.752	>0.05
3	structure_NP	0.077	0.036	2.139	<0.05^*^
4	structure_VP	−0.035	0.037	−0.943	>0.05
5	structure_CL	0.005	0.036	0.146	>0.05
6	function_DO	0.038	0.035	1.086	>0.05
7	function_RE	−0.107	0.035	−3.054	<0.01^**^
8	function_SE	0.100	0.036	2.751	<0.01^**^
9	MI	0.427	0.039	10.958	<0.001^***^
10	frequency	0.037	0.032	1.168	>0.05
11	structure_VP × MI	−0.068	0.028	−2.395	<0.05^*^
12	structure_NP × MI	−0.065	0.030	−2.161	<0.05^*^
13	structure_PP × frequency	−0.072	0.030	−2.445	<0.05^*^

PP (prepositional phrase), NP (noun phrase), VP (verb phrase), CL (clause), DO (discourse organizing), RE (referential expression), SE (stance expression), frequency (mean frequency in COCA), MI (mean Mutual Information in COCA).

* *p*< 0.05;

** *p* < 0.01;

*** *p* < 0.001.

#### Main effects in the best model (model 13)

Mean MI in COCA of nativelike 4-word sequences in essays produced the largest impact on essay scores: one unit higher mean MI in COCA associated to nativelike 4-word sequences led to 0.427 unit gains in essay score (estimate = 0.427, *p* < 0.001). To uncover sequences in high rated essays characterized by high MI, 20 4-word sequences characterized by highest MI in COCA in essays rated as high (between 20 and 24 points) and 20 such sequences in essays rated as low (between 10 and 14 points) were analyzed and compared across proficiency levels. Eng-Editor (Jin et al., 2021), an online lexical sophistication and syntactic complexity analyzer,^2^ was employed to analyze words embedded within these sequences. Eng-Editor reports at which level of Chinese EFL syllabus the query words were taught. As Table 4 illustrates, in high-rated essays nine sequences comprised one or more words taught in senior high level (e.g., *impressed, denying*), and two sequences comprised word (*rid*) from college level. In contrast, in low-rated essays only *as I am concerned* consisted of word (*concerned*) from senior high level. All other words (e.g., *lot*) were drawn from junior high level or primary level. In short, the results revealed that in senior high students’ English essays the correct use of 4-word sequences composed of the pivot words from senior high level or above (i.e., college level) were linked to raters’ favorable evaluation of essay quality.

**Table 4:** 4-Word sequences characterized by high MI in high- and low-rated essays.

	Higher essays	MI	Lower essays	MI
1	Goes without saying that	20.86	As I am **concerned**	17.15
2	Was deeply **impressed** by	19.95	Want to go somewhere	16.55
3	There is no **denying**	19.67	A lot of people	16.23
4	Was deeply touched by	18.80	All over the country	16.01
5	A far-reaching **impact** on	18.45	She had never married	15.46
6	Looked down upon by	18.22	Is a good thing	14.68
7	Can make a difference	18.02	She wanted to go	14.61
8	At the same time	17.97	In our daily life	14.02
9	A **positive** effect on	17.89	Find a good job	13.81
10	Spare no **effort** to	17.79	She spent more than	13.60
11	When it comes to	17.60	As we all know	13.09
12	Can get *rid* of	17.59	Tells us that the	13.01
13	To achieve their dreams	17.25	Travel all over the	12.99
14	Are more **likely** to	17.07	Invited to the white	12.98
15	Which **enables** them to	16.92	Because they have no	12.96
16	They are more **likely**	16.63	Have a better life	12.89
17	I am willing to	16.46	Students will learn to	12.67
18	Get *rid* of the	16.10	Some of them are	12.57
19	Makes a great difference	16.03	Do the same things	12.05
20	A large **sum** of	15.91	A good way to	12.03

The words in bold are from the senior high level (grades 10–12), the words in italics are from college level, the other words are from junior high level (grades 7–9) or primary level.

The best model (model 13) also revealed the link of higher essay scores to more stance sequences. Follow-up log likelihood ratio test showed that eight stance sequences shown in Table 5 appeared significantly more in essays rated as high (between 20 and 24 points) than in essays rated as low (between 10 and 14 points).

**Table 5:** 4-Word stance sequences appearing more in high-rated essays.

4-Word stance sequences	Raw frequency (pmw) in essays				
	High essays	Low essays	LL	*p*	Log ratio
There is no doubt	32(484)	12(249)	4.21	<0.05	0.96
There is no denying	17(257)	0	18.62	<0.0001	4.63
We are supposed to	15(227)	0	16.43	<0.0001	4.45
I am willing to	12(181)	0	13.14	<0.001	4.13
Are more likely to	11(166)	0	12.05	<0.001	4.00
Students are likely to	11(166)	0	12.05	<0.001	4.00
They are able to	10(151)	0	10.95	<0.001	3.87
Are in need of	10(151)	0	10.95	<0.001	3.87

pmw = per million words.

A close examination of these sequences revealed two important findings. First, advanced writers in high rated essays used two booster devices, *there is no doubt* and *there is no denying*, more frequently than low proficient writers did. Specifically, *there is no denying* appeared 17 times in high rated essays, but it did not appear in low rated essays. Additionally, *there is no doubt* appeared 32 times (pmw = 484) in high rated essays, outnumbering its frequency (12 times, pmw = 249) in low rated essays. These boosters established a convicted authorial stance who were fully committed to their arguments (Hyland, 2005), as Example 1 illustrates:

**Example 1**:

**There is no denying** the fact that her selfless donation makes positive difference to those who receive her help (high rated essay).

The other finding was that advanced writers in high rated essays also used hedges (e.g., *we are supposed to, are more likely to*, *students are likely to*) more frequently to express uncategorical statements than lower level writers did. For example, *we are supposed to* appeared 15 times in high rated essays, but it did not appear in low essays. In high rated essays, *we are supposed to* reduced degree of obligation on readers (inclusive ‘we’), and thus conveyed more respect to readers (Hyland, 2005) than unhedged expressions (e.g., *we should/need to pass on this traditional virtue to the young generation*), as Example 2 illustrates:

**Example 2**:

I think her donation action should be highly thought of by us and **we are supposed to** pass on this traditional virtue to the young generation. (high rated essay).

Another two hedging devices, *are more likely to* and *students are likely to*, appeared 11 times, respectively, in high rated essays. Like *we are supposed to*, they did not occur in low essays. In high rated essays, they withheld writers’ strength of argument regarding the effect of Miss McCarty’s donation on students, signaling that writers accepted different opinions, as in Example 3. They contributed to the construction of a cautious writer image who advanced deliberated arguments to accommodate other opinions (see Hyland, 2010).

**Example 3**:

Inspired by the support of Miss McCarty, the **students are likely to** become more proud and grateful, resulting in contributing to the society in the future. (high-rated essay).

Furthermore, the best model (model 13) also supported the link of higher essay scores to more noun phrase sequences. Follow-up log likelihood ratio tests revealed that nine noun phrase sequences (see Table 6) in high rated essays (between 20 and 24 points) were significantly more frequent than in low rated essays (between 10 and 14 points). Additionally, the stages of their structural patterns appeared in the well-attested five-stage developments of grammatical complexity (Biber et al., 2011) were noted in the last column.

**Table 6:** 4-Word NP sequences appearing more in high-rated essays.

4-Word NP sequences	Raw frequency (pmw) in essays					
	High essays	Low essays	LL	*p*	Log ratio	Develop-mental stage
My point of view	33(499)	9(187)	8.04	<0.01	1.42	3
The help of her	14(212)	0	15.33	<0.0001	4.35	3
A great influence on	13(196)	1(21)	8.76	<0.01	3.24	4
A helping hand to	13(196)	0	14.24	<0.001	4.24	2
Effect on the students	12(181)	0	7.82	<0.01	4.13	3
A large sum of	12(181)	0	13.14	<0.001	4.13	3
The lack of money	9(136)	1(21)	5.08	<0.05	2.71	3
The importance of helping	8(121)	0	8.76	<0.01	3.54	5
More and more students	8(121)	0	8.76	<0.01	3.54	2

Among these nine sequences, the structural patterns of seven sequences occurred in stage 3 or higher. Moreover, five (*my point of view*, *the help of her*, *a large sum of*, *the lack of money*, *the importance of helping*) comprised *of* phrase as post-modifier, and another two (*a great influence on*, *effect on the students*) comprised other prepositional phrase as post-modifier. These two structural patterns are characteristic of academic prose (Biber et al., 1999).

Finally, the link between more referential sequences and lower essay scores shown in the best model was in part due to that lower essays contained more sequences copied from the reading passage (see Appendix 1). As Table 7 illustrates, the overall frequency of copied 4-word sequences in low rated essays (between 10 and 14 points) was significantly higher than that in high rated essays (between 20 and 24 points; LL = 28.30, *p* < 0.0001, LR = 1.44).

**Table 7:** 4-Word referential sequences borrowed from passage.

4-Word referential sequences	Raw frequency (pmw)				
	Low essays	High essays	LL	*p*	Log ratio
A large amount of	42 (871)	35 (529)	4.76	*p* < 0.05	0.72
All over the country	9 (187)	0	15.54	*p* < 0.0001	4.63
In the sixth grade	10 (207)	1 (15)	11.66	*p* < 0.001	3.78
Only a few dollars	13 (270)	4 (60)	8.28	*p* < 0.01	2.16
When she wanted to	5 (104)	0	8.64	*p* < 0.01	3.78
Total	79 (1638)	40 (605)	28.30	*p* < 0.0001	1.44

To summarize, the results from GLMM revealed that in the best fit model predicting essay scores the main effects associated to nativelike 4-word sequences giving rise to higher essay scores were their higher mean MI in COCA, more noun phrase sequences and more stance sequences, as well as fewer referential sequences. Additionally, the results of supplementary log likelihood ratio tests and qualitative analyses on sample sequences from high rated essays and low-rated essays uncovered important characteristics of nativelike 4-word sequences in young English learner essays that were linked to raters’ favorable judgment of their quality. These are correct use of 4-word sequences composed of the pivot words from senior high level or above, more complex noun phrase patterns from stage 3 or higher in developmental patterns of grammatical complexity (see Biber et al., 2011) which characterized formal academic prose, using booster and hedge devices to establish adequate authorial stance, and fewer expressions copied from source passage.

#### Interaction effects in the best model (model 13)

**Figure 2:**
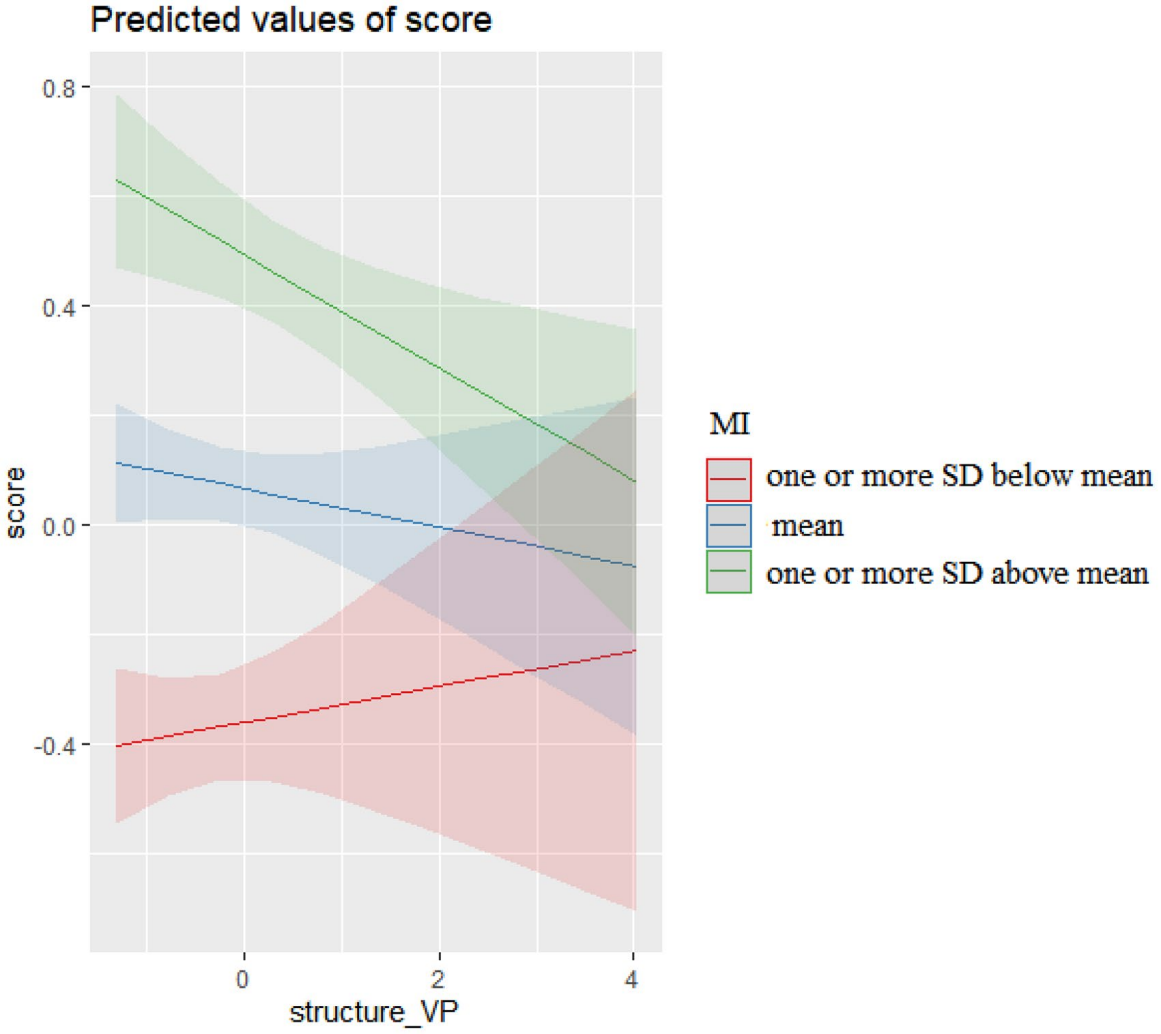
The interaction effects of VP sequences and MI on scores.

The interactions of verb phrase (VP) sequences and their MI in COCA, presented in Figure 2, show that VP sequences characterized by different levels of MI produced differential effects on essay scores. More VP sequences characterized by lower MI (one or more SD below mean) resulted in higher essay scores, but more VP sequences characterized by mean MI or higher (one or more SD above mean) resulted in lower essay scores.

Inspired by *Longman Grammar of Spoken and Written English* (LGSWE, Biber et al., 1999), the structural patterns of VP sequences in essays were categorized into “patterns more widely used in conversation,” “patterns more widely used in academic prose,” and “patterns used in both registers” (see LGSWE, pp.996–997). The differential effects of VP sequences characterized by varied levels of MI on essay scores were due to whether their structural patterns were characteristic of academic or spoken registers. As Table 8 shows, among the VP sequences characterized by lower MI (one or more SD below mean), 31.84% were “patterns more widely used in academic prose” and 60.79% were “patterns used in both academic and spoken registers.” In contrast, among the VP sequences characterized by mean MI or higher, 58.05% were patterns more widely used in conversation. The level of formality of argumentative essays is similar to that of academic prose. Therefore, using VP sequences associated to conversation may cause raters to undervalue essay quality.

**Table 8:** Distribution of nativelike VP sequences in different MI levels.

	Mean and one or more SD above mean		One or more SD below mean			
4-Word VP sequences	Number	Proportion	Number	Proportion	LL	Log ratio
Patterns more widely used in conversation						
(auxiliary +) active verb (+)	739	58.05%	28	7.37%	228.07^****^	2.98
(e.g., *help the people in)*						
Patterns more widely used in academic prose						
(1) copula be+NP/adj	93	7.31%	81	21.32%	−46.37^****^	−1.54
(e.g., *are the hope of)*						
(2) passive verb+PP fragment	64	5.03%	10	2.63%	4.23^*^	0.93
(e.g., *been out of the)*						
(3) (verb+) *that*-clause fragment	17	1.34%	30	7.89%	−35.58^****^	−2.56
(e.g., *believe that the students)*						
Subtotal	174	13.67%	121	31.84%	−47.30^****^	−1.22
Patterns used in both registers						
(verb/adjective+)*to*-clause fragment	360	28.28%	231	60.79%	−76.38^****^	−1.1
(e.g., *to help students in)*						

* denotes *p* < 0.05;

** denotes *p* < 0.01;

*** denotes *p* < 0.001;

**** denotes *p* < 0.0001.

**Figure 3:**
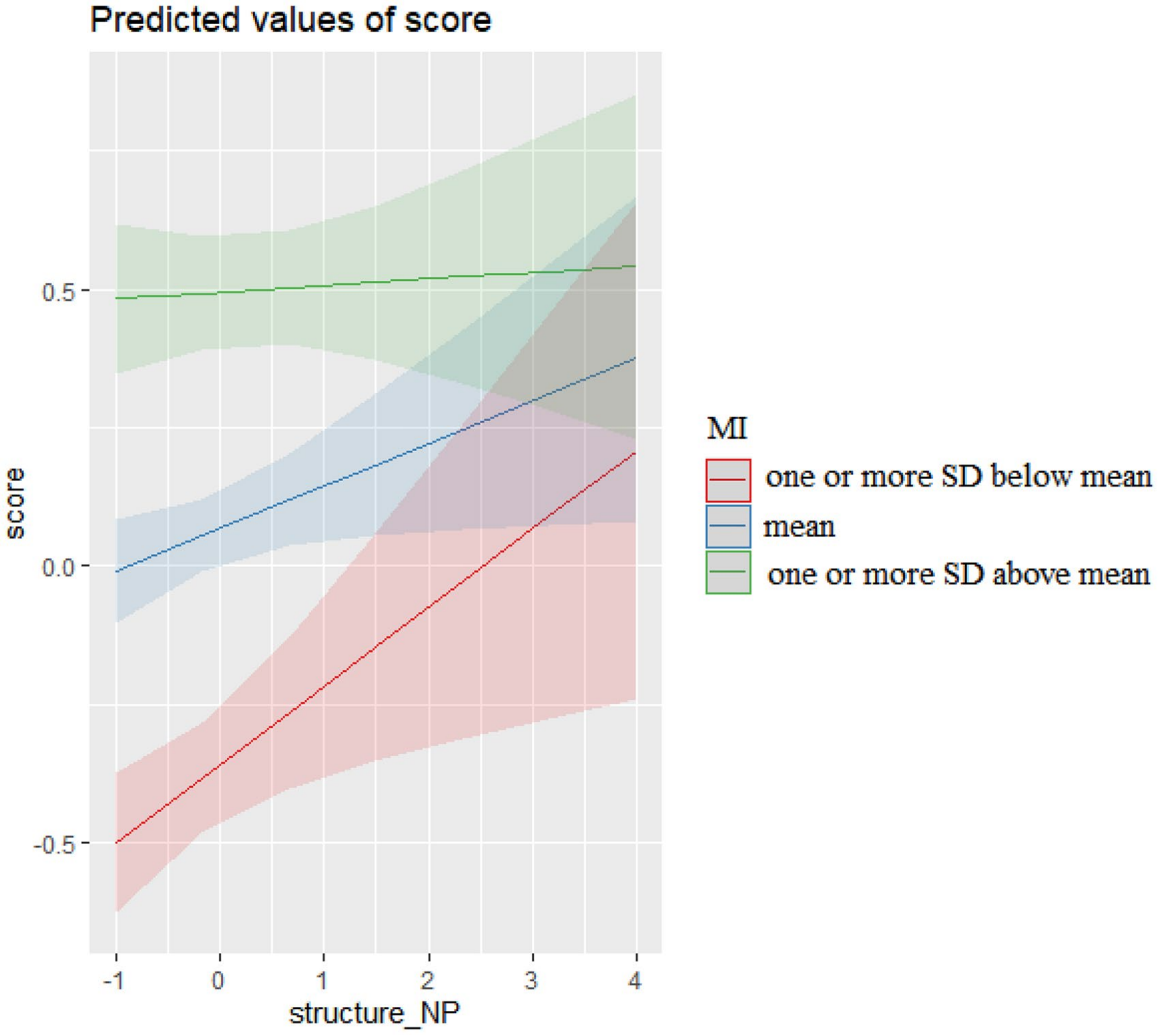
The interaction effects of NP sequences and MI on essay scores.

The interactions of noun phrase (NP) sequences and their MI in COCA, presented in Figure 3, show that more NP sequences, regardless of their MI, resulted in higher essay scores. However, their facilitative effects on essay scores were more pronounced when their MI were lower.

The differential effects of NP sequences characterized by varied levels of MI on scores were also associated to whether their structural patterns were characteristic of academic or spoken registers. As Table 9 shows, a great majority of 4-word NP sequences in essays were more widely used in academic prose (94.09% in higher level of MI, 96.86% in mean and lower level of MI), explaining their facilitative effects on score gains in all MI levels. However, more 4-word NP sequences characterized by higher level of MI (one or more SD above mean) were associated to conversation (LL = 4.79, *p* < 0.05, LR = 0.91) and they weakened the overall facilitative effects on essay scores.

**Table 9:** Distribution of structural patterns of NP sequences in different MI levels.

	One or more SD above mean		Mean and one or more SD below mean			
4-Word NP sequences	Number	proportion	Number	proportion	LL	Log ratio
*Patterns more widely used in academic prose*						
(1) noun phrase with *of*-phrase fragment (e.g.*, the hope of our*)	462	66.57%	56	11.72%	229.75^****^	2.51
(2) noun phrase with other post-modifier fragment	191	27.52%	407	85.15%	−181.01^****^	−1.63
(e.g.*, a positive effect on*)						
subtotal	653	94.09%	463	96.86%	−0.23	−0.04
*Patterns more widely used in conversation*						
(*and+*)NP (e.g.*, a better life and*)	41	5.91%	15	3.14%	4.79^*^	0.91

* denotes *p* < 0.05;

** denotes *p* < 0.01;

*** denotes *p* < 0.001;

**** denotes *p* < 0.0001.

**Figure 4:**
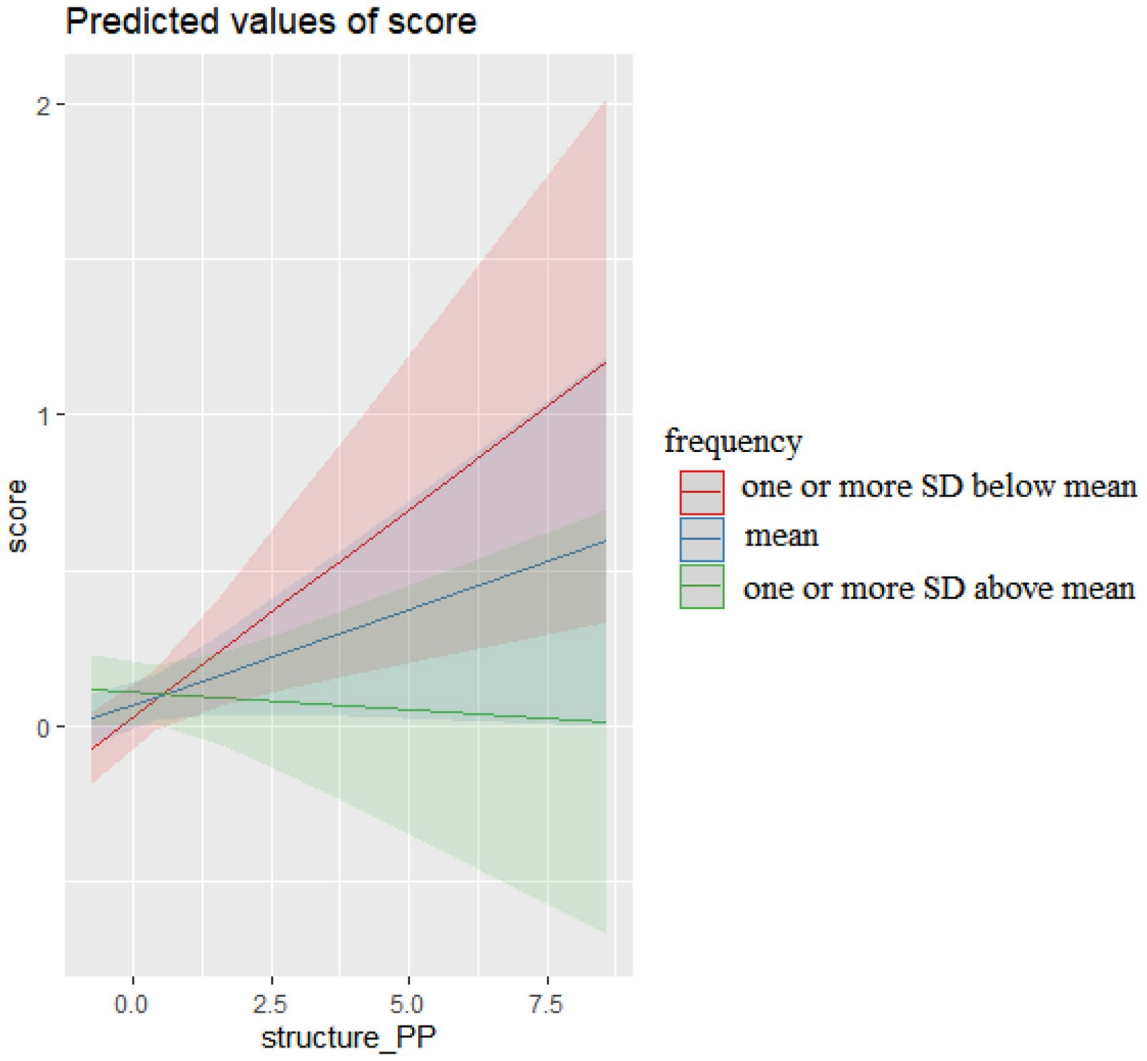
The interaction effects of PP sequences and frequency on essay scores.

Finally, the interactions of prepositional phrase (PP) sequences and their frequency in COCA, presented in Figure 4, reveal that PP sequences characterized by different levels of frequency also produced differential effects on essay scores.

More PP sequences characterized by mean frequency or lower (one or more SD below mean) in COCA resulted in higher essay scores. However, more PP sequences characterized by higher frequency (one or more SD above mean) in COCA resulted in lower essay scores. This was due to that students frequently committed grammatical errors or functional misuse when they used three of these high frequency sequences presented in Table 10.

**Table 10:** Misused 4-Word PP sequences characterized by high frequency in COCA.

4-Word PP sequences (frequency in COCA)	Number of misused examples	Total occurrence in essays	Error rates
On the other hand (19077)	124	140	88.57%
All over the world (3748)	8	25	32.00%
With the help of (3808)	13	43	30.23%

The most frequently misused 4-word sequence was *on the other hand.* Of its 140 occurrences in the essay corpus, 124 was misused as an additive device rather than as a contrastive marker, as in example 4. It confirmed the research finding of Chen and Baker (2014) as well as Bychkovska and Lee (2017) that Chinese learners of English frequently misused *on the other hand*.

**Example 4**:

I think her donation have big effect for received students, as for the poor students, they need financial help to support their schooling, it can help students without worry about matter to focus their study. On the other hand, it can help the students how to thankful the people or help themselves person, when they become an adult, they will help others. (score:10)

Additionally, *all over the world* was frequently misused as object of verb in student essays, as in:

So, Miss McCarty's donation action is not only affect the students but affect all over the world. (score: 16)

Finally, in low rated essays, the subjective case “*she*” was incorrectly used after “*with the help of*,” as in:

*With the help of she is for students the clearly needed financial help. (score: 10).*

## Discussion

This study was the first to investigate the synergistic effects of MWSs structure, function, frequency and association on raters’ evaluations of essay quality. It also enriched an emerging line of research on MWSs in essays composed by adolescent English learners and their relationships to writing quality. Two novel findings were generated from this study. First, MI-based association of nativelike 4-word sequences performed better than their frequencies in COCA, their structures as well as their functions in gauging proficiency ratings of high school English learner essays (see Table 3). It resonated with earlier research finding that underscored the predictive powers of MI-based association measure for gauging essay ratings (Bestgen and Granger, 2014; Bestgen, 2017; Paquot, 2019), but also it contributed to a better understanding of the superior predictive powers of MI-based association measure over other phraseological measures pertaining to frequency, structures as well as functions in gauging English essay quality. It was in line with the research finding of Paquot (2019) that MI-based phraseological association measure performed better than syntactic complexity measures to discriminate upper-intermediate and advanced English essays. The other novel finding was that essay scores were significantly linked to the interaction of verb phrase sequences and their MI in COCA, the interaction of noun phrase sequences and their MI in COCA, as well as the interaction of prepositional phrase sequences and their frequency in COCA (see Table 3). It filled the gap of lack of empirical research investigating how MWSs structures and their statistical features interact, as well as revealed their interaction effects on raters’ evaluations of essay quality. Furthermore, supplementary analyses for this finding have revealed the moderating effect of MI on patterns of verb phrase sequences and noun phrase sequences associated to academic prose and conversation (Tables 8, 9), as well as confirmed the moderating effect of MI on the relationship of verb phrase sequences and noun phrase sequences to essay scores (Figures 2, 3). These findings have improved our understanding of the intricate interconnections among phraseological structures, their MI and their level of formality associated to formal and informal discourse on one hand, and their joint effects on raters’ judgement of essay quality on the other hand.

Moreover, several findings from this study also inform better pedagogy of MWSs in the senior high EFL context. First, while several multiword lists have been developed to inform university students what idiomatic academic phrases they can use when writing academic assignments (e.g., Academic Formulas List (Simpson-Vlach and Ellis, 2010), Academic Collocation List (Ackermann and Chen, 2013), few efforts have been made by researchers to develop multiword list to inform English teaching and learning at the secondary level (and below). The research findings from the present study inform how to develop a pedagogically useful multiword list that specifically assist senior high (grades 10–12) English learners in the following ways. The finding that the best predictor model (model 13) can explain a fairly large proportion (marginal R^2^ = 0.260) of variance of essay scores shows that nativelike 4-word sequences in COCA, which can be accessed from COCA N-gram list,^3^ have great pedagogical values. Moreover, to further refine COCA 4-gram list to make them more relevant to and useful for senior high English learners, other research findings from this study underscored two useful parameters to serve this purpose. The first parameter is 4-word sequences in COCA composed of one or more word from senior high level textbook. This parameter is supported by the link of higher essay scores to nativelike 4-word sequences composed of one or more words from senior high level English curriculum (see section 4.2.1). Another parameter is associated to MI. The link between higher mean MI of nativelike 4-word sequences in essays and higher essay scores supports that the 4-word sequences in COCA that are composed of one or more word from senior high level textbook as well as characterized by high MI in COCA are better candidate items in a multiword list that assist senior high learners of English.

Second, the present study has established a novel finding that in student essays the levels of formality of nativelike 4-word VP sequences and NP sequences associated to academic prose and conversation were moderated by their MI in COCA. More nativelike 4-word VP sequences characterized by higher MI were associated to conversation (see Table 8), while more nativelike 4-word NP sequences characterized by higher MI were associated to academic prose (see Table 9). Pedagogically, these findings indicate that, in teaching the language features associated to argumentative essays in the senior high EFL contexts, teachers can not only draw on the 4-grams list in COCA composed of one or more word from senior high level textbook as well as characterized by high MI in COCA, they also need to emphasize which items in the above list exhibit structural patterns characterizing formal persuasive discourse (e.g., passive verb+PP fragment; noun phrase + *of* phrase fragment), as illustrated in Tables 8, 9. Additionally, *Longman Grammar of Spoken and Written English* (Biber et al., 1999) provides a comprehensive summary of 12 structural patterns characterizing academic prose (p1014-1,015) and lists the multiword sequences associated to these structural patterns (p1015-1,024). English teachers may draw on some of these structural patterns and their associated multiword sequences when they teach the language features associated to English argumentative essays.

Third, the link of high school English learners’ higher-rated essays to more complex noun phrase patterns from stage 3 or higher in the five-staged developments of grammatical complexity (see Biber et al., 2011) clearly indicates that noun phrases of higher structural complexity are closely associated to a higher level of formality characterizing argumentative essays and thus to higher essay scores. This finding supports that teaching complex noun phrase patterns associated to stage 3 or higher in the five-staged developments of grammatical complexity is important not only for college students to facilitate academic reading and writing in English (Lan et al., 2019), but also for high school English learners when they write argumentative essays. On the other hand, the result that more clause-based nativelike 4-word sequences were not linked to higher essay scores (Estimate = 0.005, *p* > 0.05, see Table 3) indicate that writing more dependent clauses in argumentative essays had little effect on increasing essay scores, as dependent clauses characterize conversation (Biber et al., 2011) and writing more dependent clauses in argumentative essays make essays become more like conversation.

Finally, the finding that advanced writers in higher-rated essays employed more sequences associated to boosters to construct confident stance as well as more sequences associated to hedges to construct accommodating stance underscores that teachers should raise students’ attentions to the rhetorical effects of stance expressions and their effects on essay quality. It was in line with accumulated research findings supporting the link of appropriate stance-taking to essay quality (Aull and Lancaster, 2014; Lancaster, 2014, 2016; Bruce, 2016). The implication is that when teaching language features associated to well-written English argumentative essays, teachers should include metadiscourse devices, such as boosters and hedges, at both single word level and multiword level (see Hyland, 2005: 218–224; Ho and Li, 2018: 67–68), because they play a crucial role to facilitate writer-reader interaction, construction of appropriate writer stance, as well as better strength of persuasion (Jiang, 2015; Ho and Li, 2018). Moreover, bringing the instructions on metadiscourse devices and their rhetorical effects into the argumentative essay course will complement other parts of the course that focus on the structural characterizations of this type of essay (e.g., frequent use of complex noun phrase patterns from stage 3 or higher expounded in Biber et al., 2011).

## Conclusion

Drawing on 900 rated argumentative essays composed by Chinese high school students in National Matriculation Test, the present study employed CollGram to automatically identify the nativelike 4-word sequences in these essays and to analyze their frequency and Mutual Information (MI) scores in COCA. The structures and functions of frequent nativelike 4-word sequences were also analyzed manually. We then investigated their synergistic effects on essay scores by constructing a serial of Generalized Linear Mixed-effect Models (GLMM) along with AIC-based comparisons and Maximum Likelihood test of significance among models to identify the best model in which the structures and functions of these nativelike 4-word sequences, their frequency and MI in COCA as well as their interactions predicted essay scores. The results from the best model (model 13 in Table 3) revealed that the main effects associated to nativelike 4-word sequences giving rise to higher essay scores were their higher mean MI in COCA, more noun phrase sequences and more stance sequences, as well as fewer referential sequences. Additionally, the interaction of prepositional phrase sequences and their frequency in COCA affected essay scores, so did the interaction of verb phrase sequences and their MI in COCA, as well as the interaction of noun phrase sequences and their MI in COCA.

Additionally, the supplementary qualitative analyses of sequences associated to the main effects in the best model have revealed important characteristics of nativelike 4-word sequences in English essays that were linked to raters’ favorable judgment of their quality. These are correct use of 4-word sequences composed of one or more words from senior high level textbook or above, more complex noun phrase patterns from stage 3 or higher in developmental patterns of grammatical complexity (see Biber et al., 2011), more booster and hedge devices to establish appropriate authorial stance, and fewer expressions copied from source passage. Moreover, qualitative analyses of the interaction of nativelike 4-word verb phrase sequences or noun phrase sequences and their MI in COCA have revealed that these sequences characterized by different levels of their MI in COCA exhibited different structural patterns associated to academic prose and conversation (see Tables 8, 9). Finally, qualitative analyses of the interaction of nativelike 4-word prepositional phrase sequences and their frequency in COCA have revealed that three 4-word prepositional phrases (*on the other hand, all over the world, with the help of*) characterized by high frequency in COCA were misused by young English learners in a high proportion of essays in our dataset.

Overall, the research findings from this study have underscored how analyses of statistical features of MWSs and analyses of their structures and functions can be combined to produce a more wide-ranging investigation into multiple aspects of MWSs in L2 writing and to gain deeper insights into their complex interactions and synergistic effects on writing quality. This study, however, has the following limitations. We only investigated nativelike contiguous 4-word sequences, but did not systematically examine discontinuous MWSs (e.g., *p*-frames) or erroneous phraseology (except several misused prepositional phrases). Future studies can draw on CollGram (see footnote 1) to extract MWSs in student essays that did not appear in COCA, analyze patterns associated to unidiomatic MWSs, and establish unidiomatic phrase profiles at various English proficiency levels. Second, we only analyzed essays written on one topic (see Appendix 1), and topic may influence multiword sequences used in these essays. Future study may draw on essays from different topics characterized by different levels of topic familiarity or cognitive difficulty, and investigate the synergistic effects of MWSs structure, function and their statistical features on essay scores under different task conditions.

## Data availability statement

The raw data supporting the conclusions of this article will be made available by the authors, without undue reservation.

## Author contributions

YL is responsible for conceptualization and writing up the paper. AF is responsible for statistical analyses. All authors contributed to the article and approved the submitted version.

## Funding

This research was supported by National Social Science Fund of China (21BYY187) awarded to YL.

## Conflict of interest

The authors declare that the research was conducted in the absence of any commercial or financial relationships that could be construed as a potential conflict of interest.

## Publisher’s note

All claims expressed in this article are solely those of the authors and do not necessarily represent those of their affiliated organizations, or those of the publisher, the editors and the reviewers. Any product that may be evaluated in this article, or claim that may be made by its manufacturer, is not guaranteed or endorsed by the publisher.

## Footnotes

^1^ http://collgram.pja.edu.pl/Default

^2^ https://www.languagedata.net/tester/

^3^ https://www.ngrams.info

## Appendix 1 The writing task of 2014 Chinese National Matriculation English Test (Guangdong version) (Reproduced with permission):

Integrated task (25 points):

### Read the following passage, and follow the instructions given below the passage to write an English essay containing approximately 150 words.

Oseola McCarthy spent more than 75 years washing and ironing othe people’s clothes. As a laundry woman, she was paid only a few dollars each time. Certainly nobody would consider her rich, so they were all amazed when Miss McCarthy decided to donate 150, 000 dollars to the University of Southern Mississippi. She could save such a large amount of money because she lived a very simple life. She never learned to drive, and when she wanted to go somewhere, she just walked. She never flew to anywhere till the donation, and in 50 years she had been out of south only once. The house in which she lived was also a rather modest one her last uncle left her. Only after she became known in America did she begin to travel all over the country. Since then, she had been the subject of many interviews and articles and was even invited to the White House. Her donation was for students who clearly needed financial help. She herself left school in the sixth grade and had never married or had children. She said to the reporters that the idea of helping somebody’s child go to college gave her much pleasure.

### Write an essay covering the following contents:

1. Use approximately 30 words to summarize the main ideas of the above passage.

2. Use approximately 120 words to express your opinions on Miss McCarthy’s donation, including (1) how you think of her donation, (2) the impact on students who received her support, (3) If you were to donate, who would be the beneficiary and explain reasons.

### Other requirements:

1. You can draw on real experiences or made-up stories, or refer to information included in the above passage. Copying the sentences from the passage is not allowed.

2. Your essay should not contain your real names and your schools.
